# R(+)-Baclofen, but Not S(−)-Baclofen, Alters Alcohol Self-Administration in Alcohol-Preferring Rats

**DOI:** 10.3389/fpsyt.2016.00068

**Published:** 2016-04-18

**Authors:** Irene Lorrai, Paola Maccioni, Gian Luigi Gessa, Giancarlo Colombo

**Affiliations:** ^1^Neuroscience Institute, National Research Council of Italy - Cagliari Section, Monserrato, Italy

**Keywords:** (±)-baclofen, R(+)-baclofen, S(−)-baclofen, GABA_B_ receptor, operant oral alcohol self-administration, Sardinian alcohol-preferring rats

## Abstract

Racemic baclofen [(±)-baclofen] has repeatedly been reported to suppress several ­alcohol-motivated behaviors, including alcohol drinking and alcohol ­self-administration, in rats and mice. Recent data suggested that baclofen may have bidirectional, stereospecific effects, with the more active enantiomer, R(+)-baclofen, suppressing alcohol intake and the less active enantiomer, S(−)-baclofen, stimulating alcohol intake in mice. The present study was designed to investigate whether this enantioselectivity of baclofen effects may also extend to the reinforcing properties of alcohol in rats. To this end, selectively bred Sardinian alcohol-preferring (sP) rats were initially trained to lever respond on a fixed ratio 4 (FR4) schedule of reinforcement for alcohol (15%, v/v) in daily 30-min sessions. Once responding had stabilized, rats were tested with vehicle, (±)-baclofen (3 mg/kg), R(+)-baclofen (0.75, 1.5, and 3 mg/kg), and S(−)-baclofen (6, 12, and 24 mg/kg) under the FR4 schedule of reinforcement. Treatment with 3 mg/kg (±)-baclofen reduced the number of lever responses for alcohol and estimated amount of self-administered alcohol by approximately 60% in comparison to vehicle treatment. R(+)-baclofen was approximately twice as active as (±)-baclofen: treatment with 1.5 mg/kg R(+)-baclofen decreased both variables to an extent similar to that of the decreasing effect of 3 mg/kg (±)-baclofen. Conversely, treatment with all doses of S(−)-baclofen failed to affect alcohol self administration. These results (a) confirm that non-sedative doses of (±)-baclofen effectively suppressed the reinforcing properties of alcohol in sP rats and (b) apparently do not extend to operant alcohol self-administration in sP rats the capability of S(−)-baclofen to stimulate alcohol drinking in mice.

## Introduction

Over the last 15 years, multiple lines of experimental evidence have demonstrated, with relatively few exceptions, that treatment with non-sedative doses of the prototypic GABA_B_ receptor agonist, baclofen, suppressed several alcohol-related behaviors – including alcohol drinking, operant alcohol self-administration, reinstatement of alcohol-seeking behavior, and alcohol-induced conditioned place preference – in rats, mice, and monkeys (1–3). These data have prompted several clinical investigations, the majority of which have extended to human alcoholics the results previously collected in laboratory animals: treatment with baclofen has often been associated with suppression of alcohol consumption, craving for alcohol, and severity of alcohol withdrawal syndrome (2, 4–6), making baclofen a novel, promising therapeutic option for alcohol use disorder, with an already widespread use in France [e.g., Ref. (7–13)].

At preclinical level, a recent and important discovery in the alcohol and baclofen research field has been the observation that the suppressing effect of baclofen on alcohol intake may be enantiomer dependent. Indeed, two recent studies demonstrated that acute treatment with the two baclofen enantiomers, R(+)-baclofen, and S(−)-baclofen, resulted in clearly opposite effects on alcohol drinking in mice: equal doses of the more active enantiomer, R(+)-baclofen, suppressed alcohol intake and of the less active enantiomer, S(−)-baclofen, stimulated alcohol intake in (a) C57BL/6J mice exposed to an experimental procedure inducing binge-like drinking and (b) selectively bred high alcohol preferring (HAP) mice exposed to chronic alcohol drinking (14, 15); this differential effect was observed after both parenteral administration (15) and infusion into the shell of the nucleus accumbens (14) of the two baclofen enantiomers. Notably, all previous studies investigating the effect of baclofen on alcohol-related behaviors have been conducted using racemic baclofen [e.g., Ref. (16–30)]; to the best of our knowledge, the only exception to the use of (±)-baclofen is constituted by a study reporting the suppressing effect of R(+)-baclofen on alcohol drinking in selectively bred, alcohol-preferring University of Chile bebedoras (UChB) rats, without testing, however, ­S(−)-baclofen (31). Thus, the evidence of the enantioselective effect of baclofen on alcohol drinking (14, 15) is novel and interesting, as it might contribute toward explaining (a) the variability in magnitude of the reducing effect of (±)-baclofen on alcohol-related behaviors [from mild, though statistically significant, reductions, e.g., Ref. (23, 29), to virtually complete suppressions, e.g., Ref. (17, 20, 24, 28)] noticed among different rodent studies and (b) the increase in alcohol drinking in rats reported in a relatively small number of studies (32–35). Variability in baclofen effect on alcohol consumption and craving has also been observed in clinical studies, both among different studies [reductions (36, 37); lack of effect (38, 39)] and within the same cohort of patients (40).

The present study was designed to contribute to this new line of research, testing the effect of the two baclofen enantiomers on operant, oral alcohol self-administration in selectively bred Sardinian alcohol-preferring (sP) rats. Alcohol self-­administration in sP rats has repeatedly been demonstrated to be sensitive to pharmacological manipulation of the GABA_B_ receptor, by means of both orthosteric agonists, such as baclofen (24–26), and positive allosteric modulators (25, 26, 41–45). The study design included (a) a dose of (±)-baclofen (3 mg/kg) known to effectively reduce alcohol self-administration in sP rats (24–26), (b) doses of the more active enantiomer, R(+)-baclofen, calculated as corresponding to half of (0.75 mg/kg), equal to (1.5 mg/kg), and double (3 mg/kg) its content in the tested dose of (±)-baclofen, and (c) doses of the less active enantiomer, S(−)-baclofen, calculated as corresponding to equal to (6 mg/kg), double (12 mg/kg), and fourfold (24 mg/kg) of its content in the tested dose of (±)-baclofen.

## Materials and Methods

All experimental procedures employed in the present study were conducted in accordance with the Italian law on the “Protection of animals used for scientific reasons.”

### Animals

Male sP rats (*n* = 96) from the 88th generation, and 60 days old at the start of the study, were utilized. Rats were alcohol-naive before the start of the study. Rats were housed three per cage in standard plastic cages with wood chip bedding. The animal facility was under an inverted 12:12-h light–dark cycle (lights on at 7:00 p.m.), at a constant temperature of 22 ± 2°C and relative humidity of approximately 60%. Over the 2-week period preceding the start of the study, rats were extensively habituated to handling and intraperitoneal injections; specifically, rats received 10 daily injections (Monday to Friday over both weeks) of 2 ml/kg saline. Food pellets (Harlan, San Pietro al Natisone, Italy) and water were always available in the home cage, except as noted.

### Apparatus

Self-administration sessions were conducted in modular chambers (Med Associates, St. Albans, VT, USA) located in sound-attenuated cubicles, with fans for ventilation and background white noise. The front panel of each chamber was equipped with (a) two retractable response levers, (b) one dual-cup liquid receptacle positioned between the two levers, and (c) two stimulus lights (one green and one white) mounted above each lever. The liquid receptacle was connected by polyethylene tubes to two syringe pumps located outside the chamber. A white house light was centered at the top of the back wall of each chamber. For half of the rats, the right lever was associated with alcohol; achievement of the response requirement (RR) (a) activated the alcohol pump, resulting in the delivery of 0.1 ml of alcohol solution and (b) switched on the green light for the 2-s period of alcohol delivery; for these rats, the left lever was associated with water, and achievement of RR (a) activated the water pump, resulting in the delivery of 0.1 ml water and (b) switched on the white light for the 2-s period of water delivery. The study design was counterbalanced, so that the opposite condition was applied to the other half of the rats (left lever: alcohol; right lever: water).

### Experimental Procedure

#### Two-Bottle Choice Phase

Rats were initially exposed to the home cage two-bottle “alcohol vs. water” choice regimen with unlimited access for 24 h/day over 10 consecutive days. The alcohol solution was presented at a concentration of 10% (v/v). This initial phase was (a) part of the conventional procedure of alcohol self-administration employed in this laboratory with sP rats [e.g., Ref. (26)] and (b) conducted to allow the rats to become accustomed to the taste of alcohol and start to experience its pharmacological effects, in order to possibly shorten the subsequent auto-shaping phase once rats were introduced into the operant chambers. During this phase, daily alcohol intake and preference (defined as the percent ratio between consumed volumes of alcohol solution and water) averaged approximately 6.3 g/kg and 63%, respectively. On the last day, alcohol intake and preference averaged approximately 7.5 g/kg and 77%, respectively.

#### Shaping, Training, and Maintenance Phases

Immediately after the two-bottle choice drinking period, rats were introduced into the operant chambers and trained to lever respond for alcohol. Self-administration sessions lasted 30 min (with the sole exception of the first session that lasted 120 min) and were conducted 5 days/week (Monday to Friday) during the first half of the dark phase of the daily light–dark cycle. Rats were deprived of water during the 12-h prior to the first session in the operant chamber. Rats were initially exposed to a fixed ratio 1 (FR1) schedule of reinforcement for 10% alcohol (v/v) for four consecutive daily sessions. FR was then increased to FR2 and FR4 over four consecutive sessions. In sessions 9 and 10, the alcohol solution was presented at a final concentration of 15% (v/v). Rats were then exposed to four consecutive sessions during which the water lever alone or the alcohol lever alone was available every other day; water and alcohol were available on FR1 and FR4, respectively. From then onward, both levers were concomitantly available (maintenance phase) for a total of 30 sessions conducted with FR4 and FR1 on the alcohol and water lever, respectively. In the last five sessions of the maintenance phase, the number of lever responses for alcohol averaged 243 ± 13, 245 ± 13, 250 ± 12, 239 ± 15, and 248 ± 13 (mean ± SEM of *n* = 96 rats), respectively; estimated amount of self-administered alcohol averaged 0.94 ± 0.05, 0.94 ± 0.05, 0.99 ± 0.05, 0.92 ± 0.06, and 0.94 ± 0.05 g/kg (mean ± SEM of *n* = 96 rats), respectively.

#### Test Phase

Rats were divided into eight groups of *n* = 12 individuals, matched for body weight and number of lever responses on the alcohol lever over the last five consecutive sessions preceding the test session. Test session was conducted the day after termination of the 30-day maintenance phase. It was virtually identical to self-administration sessions of the maintenance phase (i.e., RR on the alcohol and water lever was kept at FR4 and FR1, respectively; 30-min duration). (±)-Baclofen (Sigma-Aldrich, Milan, Italy), R(+)-baclofen (Sigma-Aldrich, Milan, Italy), and S(−)-baclofen (Sigma-Aldrich, Milan, Italy) were dissolved in saline and administered intraperitoneally (injection volume: 2 ml/kg) 30 min before the start of the test session. (±)-Baclofen was administered at the dose of 3 mg/kg, according to the results of several, previous experiments demonstrating that it effectively suppressed alcohol self-administration in sP rats (24–26). ­R(+)-baclofen was administered at the doses of 0.75, 1.5, and 3 mg/kg, corresponding to half of, equal to, and double its content in the tested dose of (±)-baclofen [(±)-baclofen is indeed a 1:1 mixture of the two enantiomers]. S(−)-baclofen was administered at the doses of 6, 12, and 24 mg/kg, corresponding to equal to, double, and fourfold of its content in the tested dose of (±)-baclofen. Vehicle condition was represented by administration of an equal volume of saline.

### Variables and Data Analysis

Variables were number of responses on each lever and estimated amount of self-administered alcohol (expressed in gram per kilogram of pure alcohol; determined from the number of earned reinforcers, as careful inspections at the end of each session indicated that no alcohol was left in any receptacle). When normally distributed, data were analyzed by one-way ANOVA, followed by the Tukey’s test for *post hoc* comparisons; when not normally distributed, data were analyzed by Kruskal–Wallis test.

## Results

ANOVA indicated that drug treatment significantly affected both the number of lever responses for alcohol [*F*(7,88) = 8.79, *P* < 0.0001] and estimated amount of self-administered alcohol [*F*(7,88) = 7.24, *P* < 0.0001].

**Figure 1:**
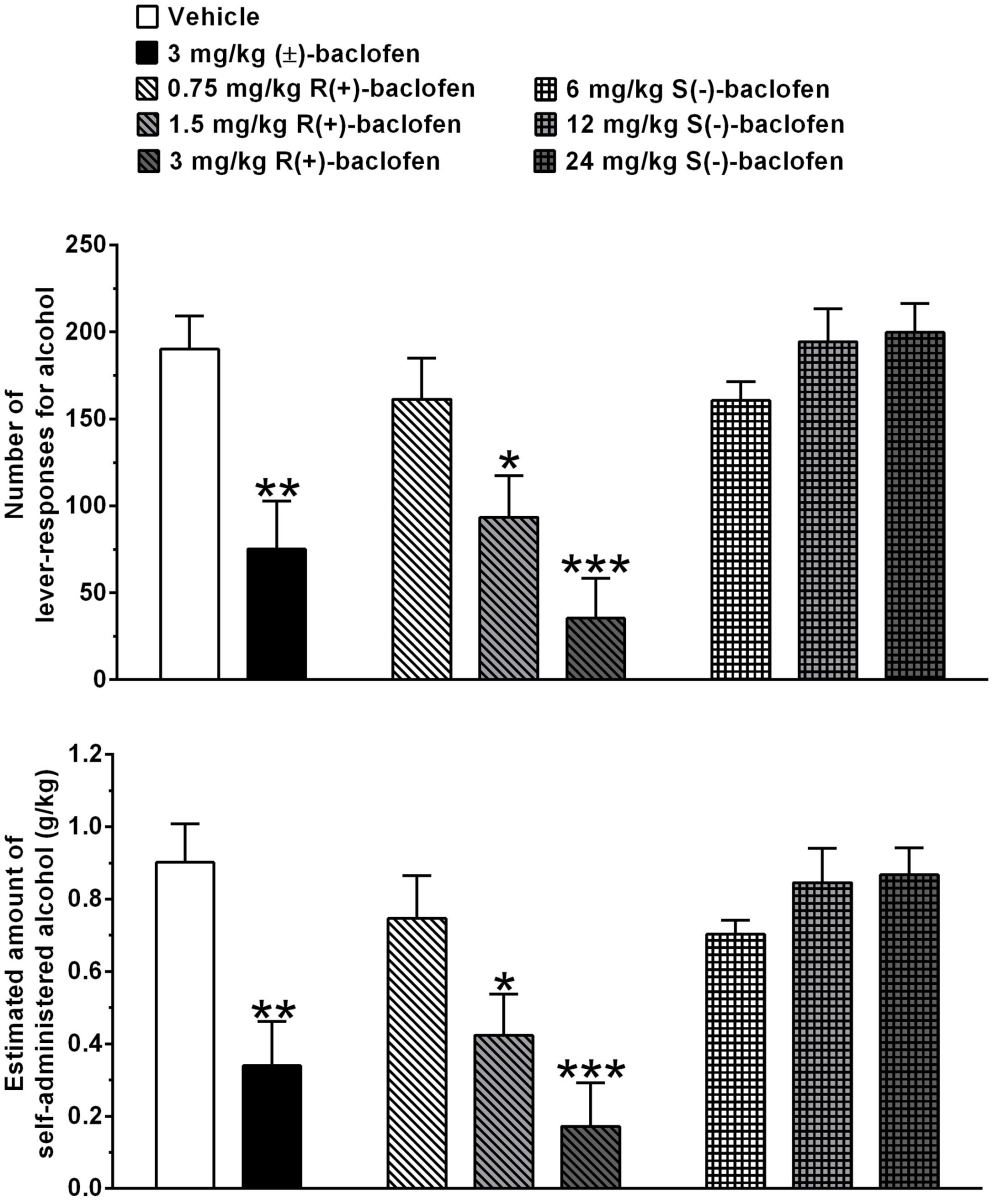
**Effect of treatment with racemic baclofen [(±)-baclofen], R(+)-baclofen, and S(−)-baclofen on number of lever responses for alcohol (top panel) and estimated amount of self-administered alcohol (bottom panel) in selectively bred Sardinian alcohol-preferring (sP) rats**. Rats were initially trained to lever respond for oral alcohol (15% v/v, in water) [fixed ratio 4 (FR4)] and water (FR1) in daily 30-min sessions. Test session was conducted under the above FR schedules of reinforcement. Each bar is the mean ± SEM of *n* = 12 rats. *P < 0.05, **P < 0.005, and ***P < 0.0001 in comparison to vehicle-treated rats (Tukey’s test).

Treatment with 3 mg/kg (±)-baclofen produced a reduction of approximately 60%, in comparison to vehicle treatment, in number of lever responses for alcohol (*P* < 0.005, Tukey’s test) (Figure 1, top panel). Treatment with 0.75, 1.5, and 3 mg/kg R(+)-baclofen produced a dose-related reduction in number of lever responses for alcohol, reaching statistical significance at the doses of 1.5 (*P* < 0.05, Tukey’s test) and 3 (*P* < 0.0001, Tukey’s test) mg/kg, in correspondence of which the magnitude of the reducing effect – in comparison to vehicle treatment – averaged approximately 50 and 80%, respectively (Figure 1, top panel). Conversely, treatment with 6, 12, and 24 mg/kg S(−)-baclofen did not alter, in comparison to vehicle treatment, the number of lever responses for alcohol (Figure 1, top panel).

Similar changes, in comparison to vehicle treatment, were recorded in the estimated amount of self-administered alcohol (Figure 1, bottom panel): (a) treatment with 3 mg/kg (± )-baclofen produced a reduction of approximately 60% in comparison to vehicle treatment (*P* < 0.005, Tukey’s test), (b) treatment with 1.5 (*P* < 0.05, Tukey’s test) and 3 (*P* < 0.0001, Tukey’s test) mg/kg R(+)-baclofen produced a reduction of approximately 55 and 80%, respectively, in comparison to vehicle treatment, and (c) treatment with 6, 12, and 24 mg/kg S(−)-baclofen was ineffective.

Kruskal–Wallis test indicated that drug treatment had no effect on number of lever responses for water [χ^2^ = 7.09, df = 7, *P* > 0.05]. The number of lever responses for water was negligible (averaging <2 per session at all treatment conditions) (data not shown).

## Discussion

**Figure 2:**
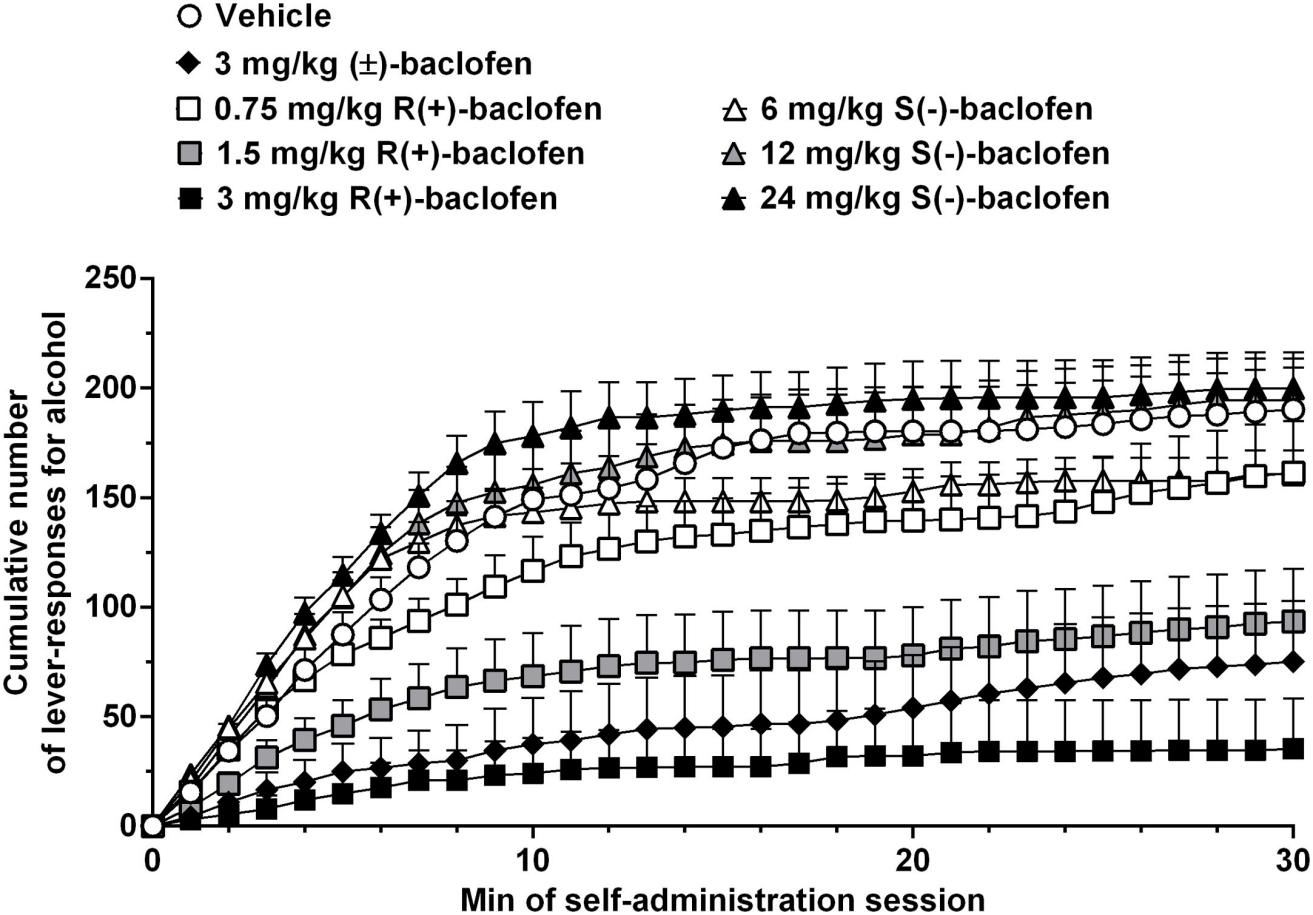
**Effect of treatment with racemic baclofen [(±)-baclofen], R(+)-baclofen, and S(−)-baclofen on cumulative response pattern of self-administration for alcohol in selectively bred Sardinian alcohol-preferring (sP) rats**. Rats were initially trained to lever respond for oral alcohol (15% v/v, in water) [fixed ratio 4 (FR4)] and water (FR1) in daily 30-min sessions. Test session was conducted under the above schedules of reinforcement. Each point is the mean ± SEM of *n* = 12 rats.

Treatment with 3 mg/kg (±)-baclofen produced a robust reduction in alcohol self-administration in alcohol-preferring sP rats: both the number of lever responses for alcohol and estimated amount of self-administered alcohol were indeed suppressed by treatment with 3 mg/kg (±)-baclofen. Additionally, analysis of cumulative response patterns indicated a markedly reduced frequency in lever responding for alcohol, over the entire first half of the self-administration session, in the rat group treated with 3 mg/kg (±)-baclofen in comparison to vehicle-treated rat group (Figure 2). These results closely replicate previous findings obtained in sP rats exposed to experimental procedures of operant, oral alcohol self-administration identical to those used in the present study (24, 26). Notably, previous experiments demonstrated that acute treatment with 3 mg/kg (±)-baclofen did not affect, even minimally, spontaneous locomotor activity in sP rats (17, 18), suggesting that the suppressive effect on alcohol self-administration observed in the present study was not due to any motor-impairing or sedative effect. Together, these data suggest that 3 mg/kg is a dose of (±)-baclofen suitable for functioning as reference when testing baclofen enantiomers in sP rats.

Racemic baclofen [(±)-baclofen] is composed of a 1:1 mixture of R- and S-configurations, with the R-enantiomer being 100–1000 times more potent, both *in vitro* [e.g., Ref. (46)] and *in vivo* [e.g., Ref. (47, 48)], than the S-enantiomer. Therefore, R-baclofen is the enantiomer on which the pharmacological activity of (±)-baclofen is based. Accordingly, in the present study, R(+)-baclofen appeared to be twice as active as (±)-baclofen: administration of 1.5 mg/kg R(+)-baclofen reduced both the number of lever responses for alcohol and estimated amount of self-administered alcohol to an extent similar to that produced by administration of 3 mg/kg (±)-baclofen. Additionally, reductions produced by 0.75 mg/kg R(+)-baclofen [corresponding to 1.5 mg/kg (±)-baclofen] and 3 mg/kg R(+)-baclofen [corresponding to 6 mg/kg (±)-baclofen] were proportionally smaller and larger, respectively, than that produced by 3 mg/kg (±)-baclofen.

Conversely, none of the three tested doses of S(−)-baclofen (6, 12, and 24 mg/kg) altered the number of lever responses for alcohol and estimated amount of self-administered alcohol, confirming its inactivity even at relatively high doses. A tendency toward a higher frequency of responding on the alcohol lever, in the rat group treated with 24 mg/kg S(−)-baclofen and during the 5–15 min time period of the self-administration session, was the only possible effect of S(−)-baclofen that could be detected (Figure 2).

Together, data collected in the present study on R(+)-baclofen and S(−)-baclofen confirm the stereospecificity of baclofen observed in several receptor-binding [e.g., Ref. (46)] and behavioral [e.g., Ref. (47)] studies. These data are also in agreement with the results of *in vivo* studies indicating that the R-enantiomer is around twice as active as (±)-baclofen [e.g., Ref. (47, 48)].

Apart from the temporarily limited increase in number of lever responses for alcohol induced by treatment with 24 mg/kg S(−)-baclofen (Figure 2), the results of the present study are apparently far from replicating the recent findings of the capability of S(−)-baclofen to effectively stimulate alcohol intake in HAP mice and binge-like drinking in C57BL/6J mice (14, 15). Notably, stimulation and reduction of alcohol drinking by S(−)-baclofen and R(+)-baclofen, respectively, occurred at the same dose (10 mg/kg, i.p.), suggesting that the two enantiomers were equally potent in exerting their opposite effects (15). Conversely, in the present study, S(−)-baclofen was ineffective even at a dose (24 mg/kg) that was 16 times higher than the minimum effective dose of R(+)-baclofen (1.5 mg/kg). A ceiling effect limiting S(−)-baclofen-induced increase in alcohol self-administration in sP rats does not seem to be a feasible explanation for the results of the present study, as several previous experiments have demonstrated that – under specific experimental conditions – sP rats can self-administer remarkably larger amounts of alcohol in even shorter self-administration sessions [e.g., Ref. (43)]. Additionally, the number of lever responses for alcohol and estimated amount of self-administered alcohol in S(−)-baclofen-treated rat groups tend to be lower than those recorded over the final period of the self-administration phase preceding the pharmacological test with baclofen, suggesting that there was room for possible increases in both variables.

However, several methodological differences may explain these discrepancies. First, the two studies reporting ­S(−)-baclofen-induced increase in alcohol drinking used mice (14, 15), whereas the present study used rats of a line selectively bred for high alcohol preference and consumption; species differences in the neural substrates mediating baclofen effects cannot be excluded. Second, the two studies reporting ­S(−)-baclofen-induced increase in alcohol drinking employed two experimental procedures of alcohol drinking [namely, two-bottle “alcohol (10%, v/v) vs. water” choice regimen with unlimited access for 24 h/day; 2-h limited access to a single alcohol (20%, v/v) bottle during the dark phase of the light–dark cycle] (14, 15), whereas the present study employed an operant procedure of oral alcohol self-administration, in which rats were required to perform a given task (lever responding) to access alcohol; alcohol drinking and alcohol self-administration are likely differentiable for a number of aspects, including the underlying neural substrates and their sensitivity to pharmacological manipulation. Experiments testing the effect of R(+)-baclofen and S(−)-baclofen in mice exposed to operant procedures of alcohol self-administration and/or rats exposed to alcohol drinking procedures might provide a contribution toward clarifying these discrepancies.

In conclusion, the results of the present study confirm several previous findings indicating that (a) non-sedative doses of (±)-baclofen effectively suppressed the reinforcing properties of alcohol [e.g., Ref. (19–21, 23–27)] and (b) R-baclofen is the active enantiomer of baclofen [e.g., Ref. (46, 47)]. Conversely, the results of the present study apparently do not extend to operant alcohol self-administration in sP rats the capability of the supposedly inactive enantiomer, S(−)-baclofen, to stimulate alcohol drinking – including binge-like drinking – in mice (14, 15): treatment with even relatively high doses of S(−)-baclofen was indeed completely ineffective on alcohol self-administration in sP rats.

## Author Contributions

IL, PM, and GC conceived the study and designed the experiment. GC managed the literature search and summaries of previous related work. IL and PM conducted the experiment and analyzed the data. GC and GG drafted the manuscript. All authors contributed to and approved the final draft of the manuscript.

## Conflict of Interest Statement

The authors declare that the research was conducted in the absence of any commercial or financial relationships that could be construed as a potential conflict of interest.
